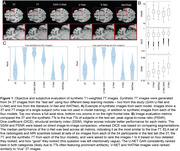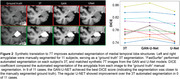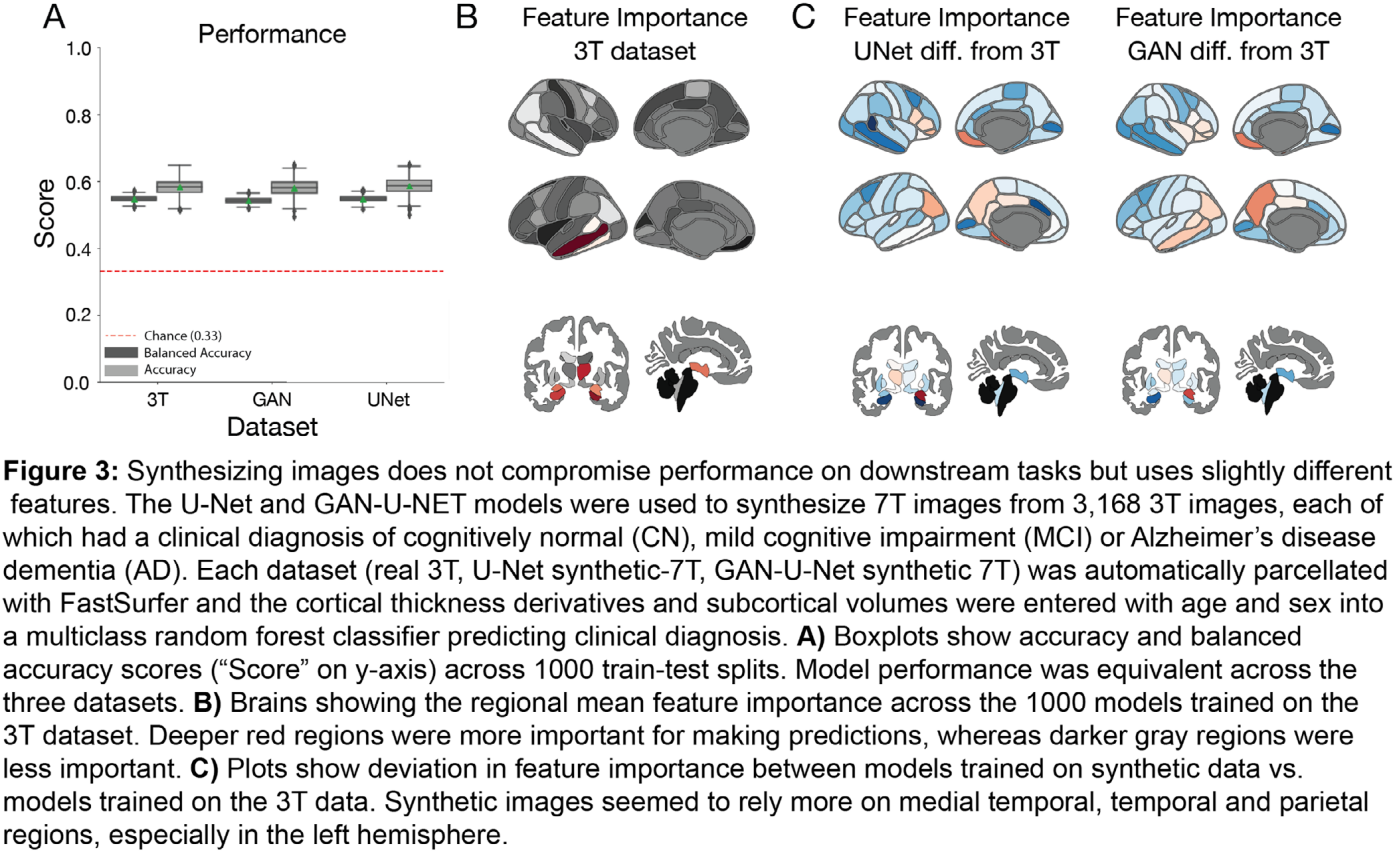# AI Superresolution: Converting T1‐weighted MRI from 3T to 7T resolution toward enhanced imaging biomarkers for Alzheimer’s disease

**DOI:** 10.1002/alz70862_109817

**Published:** 2025-12-23

**Authors:** Malo Gicquel, Gabrielle Flood, Ruoyi Zhao, Anika Wuestefeld, Nicola Spotorno, Olof Strandberg, Yu Xiao, Kalle Åström, Laura E.M. Wisse, Danielle van Westen, David Berron, Oskar Hansson, Jacob W. Vogel

**Affiliations:** ^1^ Department of Clinical Sciences Malmö, SciLifeLab, Lund University, Lund Sweden; ^2^ Centre for Mathematical Sciences, Lund University, Lund Sweden; ^3^ Clinical Memory Research Unit, Department of Clinical Sciences Malmö, Lund University, Lund Sweden; ^4^ Diagnostic Radiology, Department of Clinical Sciences Lund, Lund University, Lund Sweden; ^5^ Center for Behavioral Brain Sciences (CBBS), Otto‐von‐Guericke University Magdeburg, Magdeburg, Sachsen Anhalt Germany; ^6^ German Center for Neurodegenerative Diseases (DZNE), Magdeburg Germany

## Abstract

**Background:**

High‐resolution (7T) MRI facilitates in vivo imaging of fine anatomical structures selectively affected in Alzheimer’s disease (AD), including medial temporal lobe subregions. However, 7T data is challenging to acquire and largely unavailable in clinical settings. Here, we use deep learning to synthesize 7T resolution T1‐weighted MRI images from lower‐resolution (3T) images.

**Method:**

Paired 7T and 3T T1‐weighted images were acquired from 178 participants (134 clinically unimpaired, 48 impaired) from the Swedish BioFINDER‐2 study. To synthesize 7T‐resolution images from 3T images, we trained two models: a specialized U‐Net, and a U‐Net mixed with a generative adversarial network (U‐Net‐GAN) on 80% of the data. We evaluated model performance on the remaining 20%, compared to models from the literature (V‐Net, WATNet), using image‐based performance metrics and by surveying five blinded MRI professionals based on subjective quality. For *n* = 11 participants, amygdalae were automatically segmented with FastSurfer on 3T and synthetic‐7T images, and compared to a manually segmented “ground truth”. To assess downstream performance, FastSurfer was run on *n* = 3,168 triplets of matched 3T and AI‐generated synthetic‐7T images, and a multi‐class random forest model classifying clinical diagnosis was trained on both datasets.

**Result:**

Synthetic‐7T images were generated for images in the test set (Figure 1A). Image metrics suggested the U‐Net as the top performing model (Figure 1B), though blinded experts qualitatively rated the GAN‐U‐Net as the best looking images, exceeding even real 7T images (Figure 1C). Automated segmentations of amygdalae from the synthetic GAN‐U‐Net model were more similar to manually segmented amygdalae, compared to the original 3T they were synthesized from, in 9/11 images (Figure 2). Classification obtained modest performance (accuracy∼60%) but did not differ across real or synthetic images (Figure 3A). Synthetic image models used slightly different features for classification (Figure 3B).

**Conclusion:**

Synthetic T1‐weighted images approaching 7T resolution can be generated from 3T images, which may improve image quality and segmentation, without compromising performance in downstream tasks. This approach holds promise for better measurement of deep cortical or subcortical structures relevant to AD. Work is ongoing toward improving performance, generalizability and clinical utility.